# Exploiting Epigenetic Variations for Crop Disease Resistance Improvement

**DOI:** 10.3389/fpls.2021.692328

**Published:** 2021-06-04

**Authors:** Pengfei Zhi, Cheng Chang

**Affiliations:** College of Life Sciences, Qingdao University, Qingdao, China

**Keywords:** disease resistance, crop breeding, epigenetic variation, defense priming, DNA methylation, histone modification, chromatin assembly and remodeling, epigenetic memory

## Abstract

Pathogen infections seriously threaten plant health and global crop production. Epigenetic processes such as DNA methylation, histone post-translational modifications, chromatin assembly and remodeling play important roles in transcriptional regulation of plant defense responses and could provide a new direction to drive breeding strategies for crop disease resistance improvement. Although past decades have seen unprecedented proceedings in understanding the epigenetic mechanism of plant defense response, most of these advances were derived from studies in model plants like Arabidopsis. In this review, we highlighted the recent epigenetic studies on crop-pathogen interactions and discussed the potentials, challenges, and strategies in exploiting epigenetic variations for crop disease resistance improvement.

## Introduction

The dense monoculture of domesticated crops usually enhances the production value of cultivated land in intensive agriculture (Bruce, 2012). However, crop plants in these monocultures are particularly vulnerable to plant diseases caused by host-adapted pathogens. Therefore, developing disease-resistant crop varieties is essential to secure and enhance crop production in sustainable agriculture (Nicaise, 2017; Oliva and Quibod, 2017; Silva et al., 2018). To this end, it is vital to decipher the molecular mechanism underlying the plant host-pathogen interactions. During the long-term co-evolution with host-adapted pathogens, plants have acquired a sophisticated induced defense system to cope with pathogen infections (van der Burgh and Joosten, 2019; Zhou and Zhang, 2020). Recognition of pathogen-associated molecular patterns (PAMPs) or damage-associated molecular patterns (DAMPs) by plant pattern recognition receptors (PRRs) activates the pattern-triggered immunity (PTI), whereas detection of pathogen-secreted effectors by plant resistance (R) proteins initiates the effector-triggered immunity (ETI) (Couto and Zipfel, 2016; Jones et al., 2016; Li et al., 2016; Yu et al., 2017; Saijo et al., 2018). Although PTI and ETI differ in the magnitude and duration of downstream defense responses, they are mutually potentiated in the unified plant immunity and both associated with a massive transcriptional reprogramming of defense-related genes (Birkenbihl et al., 2017; Adachi and Tsuda, 2019; Bjornson et al., 2021; Ngou et al., 2021; Pruitt et al., 2021; Yuan et al., 2021). Increasing evidence revealed that epigenetic processes such as DNA methylation, histone post-translational modifications, chromatin assembly and remodeling govern this defense-related transcriptional reprogramming and play key roles in the regulation of crop disease resistance against a wide range of phytopathogens, including viruses, bacteria, fungi, oomycetes, netamodes, and herbivorous insects (Ding and Wang, 2015; Espinas et al., 2016; Zhu et al., 2016; Ramirez-Prado et al., 2018a,b; Wang C. et al., 2018; Alonso et al., 2019).

## Dna (De)Methylation Dynamics And Its Biological Relevance In Crop-Pathogen Interactions

As a heritable epigenetic mark, DNA methylation usually occurs at the C5 position of cytosine base in the context of CG, CHG, and CHH (H=A, C, and T) to form 5-methylcytosine (5 mC), and plays important roles in the regulation of plant development, stress adaptation and genome evolution (Colot and Rossignol, 1999; Tirnaz and Jacqueline, 2019). A recent study in liverwort *Marchantia polymorpha*, an early-diverging terrestrial plant lineage, revealed that the extensive N4 cytosine methylation is essential to *Marchantia* spermatogenesis, further expanding the scope of functional DNA methylation in plants (Walker et al., 2021). In general, DNA methylation profile is dynamically shaped by three processes involving *de novo* methylation, maintenance methylation, and active demethylation (Henderson and Jacobsen, 2007). *De novo* methylation is triggered by small RNAs via an RNA-dependent DNA methylation (RdDM) process regulated by the Domains Rearranged Methyltranferase 2 (DRM2) (Erdmann and Picard, 2020). Once established, DNA methylation could be either maintained by methyltransferase1 (MET1) and plant-specific chromomethylases (CMT2 and CMT3), or removed by DNA demethylases, including Repressor of Silencing 1 (ROS1), Demeter (DME), Demeter-Like 2 (DML2), and DML3 in Arabidopsis (Elhamamsy, 2016). Detailed mechanisms of DNA methylation and demethylation have been extensively discussed by prior reviews (Chan et al., 2005; Law and Jacobsen, 2010; Elhamamsy, 2016).

With the contribution of DNA (de)methylation mutants and advanced DNA methylation profiling techniques such as methylation-sensitive amplified fragment length polymorphism (MSAP) analysis, whole-genome bisulfite sequencing (WGBS), methylated DNA immunoprecipitation sequencing (MeDIP-seq), and methyl-CpG binding domain protein capture sequencing (MBDCap-seq), dynamics and biological functions of DNA (de)methylation in plant-pathogen interactions have been extensively studied in model and crop plants (Clark et al., 1994; Guevara et al., 2017; Li et al., 2018; Feng and Lou, 2019; Tirnaz and Jacqueline, 2019; Hsu et al., 2020). As a part of plant defense response, DNA hypomethylation is induced by pathogen infections in many plant species such as *Arabidopsis thaliana, Oryza sativa, Nicotiana tabacum, Glycine max, Brassica rapa, Citrullus lanatus*, and *Aegilops tauschii* (Dowen et al., 2012; Yu et al., 2013; Rambani et al., 2015; Kellenberger et al., 2016; López Sánchez et al., 2016; Wang C. et al., 2018; Geng et al., 2019; Sun et al., 2019; Atighi et al., 2020; Annacondia et al., 2021). A similar DNA hypomethylation is observed upon application of bacterial PAMP flg22, nematode PAMP “NemaWater,” and plant defense hormone salicylic acid (SA) in model and crop plants (Ngom et al., 2017; Atighi et al., 2020). High-resolution DNA methylation profiling revealed that this pathogen-induced DNA hypomethylation occurs in many chromatin regions proximal to defense-related genes, including promoters, gene bodies, and nearby transposable elements (TEs) (Dowen et al., 2012; Yu et al., 2013; Rambani et al., 2015; Kellenberger et al., 2016; López Sánchez et al., 2016; Ngom et al., 2017; Wang C. et al., 2018; Geng et al., 2019; Sun et al., 2019; Atighi et al., 2020; Annacondia et al., 2021). Although combined analysis of DNA methylation and gene expression revealed that this pathogen-induced DNA hypomethylation is generally correlated with transcriptional activation of proximal defense-related genes, specific regulation of defense-related gene transcription by nearby DNA hypomethylation varies among plant species and genes (Yu et al., 2013; López Sánchez et al., 2016; Geng et al., 2019; Atighi et al., 2020). For instance, chemical inhibition of DNA methylation at the promoters of rice resistance gene *XA21G* could activate the *XA21G* transcription and establish the rice resistance against bacterial blight (BB) caused by *Xanthomonas oryzae* pv. *oryzae* (*Xoo*) (Akimoto et al., 2007). In contrast, the same chemical inhibition of DNA methylation at the promoters of rice blast disease-resistance gene *Pib* compromised the *Pib* transcription and rice resistance against blast disease (Li et al., 2011). In addition to regulating nearby defense genes *in cis*, DNA (de)methylation could also regulate distant defense genes *in trans* (Tirnaz and Jacqueline, 2019). For instance, an elegant DNA methylation and gene expression analysis in the Arabidopsis hyper/hypo-methylated mutants showed that only 15% of defense-related genes induced in hypo-methylated *nrpe1* mutant and repressed in hyper-methylated *ros1* mutant were associated with a proximal TE and NRPE1- and/or ROS1-controlled DNA methylation, indicating the presence of both *cis*- and *trans*-regulation of defense-related gene transcription by DNA (de)methylation (López Sánchez et al., 2016).

In addition to these sequencing and *in silico* evidence, genetic studies also provide important implications for the involvement of DNA (de)methylation in plant defense response to pathogenic microbes (Yang et al., 2013; Annacondia et al., 2018; Diezma-Navas et al., 2019). In Arabidopsis, DNA hypomethylation mutant *nrpe1, met1-3*, and *ddc* (*drm1-2 drm2-2 cmt3-11*) exhibited potentiated resistance against the biotrophic oomycete pathogen *Hyaloperonospora arabidopsidis* (*Hpa*) and hemibiotrophic bacterial pathogen *Pseudomonas syringae* pv. *tomato* DC3000 (*Pst* DC3000) but enhanced susceptibility against necrotrophic fungal pathogen *Plectosphaerella cucumerina* (*Pc*), whereas DNA hypermethylation mutant *ros1-4* showed attenuated resistance against *Pst* DC3000 and *Hpa* but enhanced resistance against *Pc* (Dowen et al., 2012; Yu et al., 2013; López Sánchez et al., 2016). In *Aegilops tauschii*, knockdown of a *DRM2* homolog by barley stripe mosaic virus (BSMV)-based virus-induced gene silencing (VIGS) enhanced plant resistance to the fungal pathogen *Blumeria graminis* f. sp. *tritici* (*Bgt*), the causal agent of wheat powdery mildew (Table 1, Geng et al., 2019). Increasing evidence revealed that DNA (de)methylation also gets involved in the regulation of plant defense response to pests and nematodes (Leonetti and Molinari, 2020). For instance, Arabidopsis mutant plants deficient in methylation of DNA and H3K9 (*kyp*) showed increased resistance to the infestation of green peach aphid *Myzus persicae* (Annacondia et al., 2021). Similarily, rice RdDM and DDM1 mutants *dcl3, ago4, drm2*, and *ddm1* exhibited attenuated susceptibility to the infection of nematode *Meloidogyne graminicola*, confirming a central role of DNA (de)methylation in the regulation of plant defense against pathogenic microbes, pests, and nematodes (Table 1, Atighi et al., 2020). In addition, DNA (de)methylation was shown to orchestrate the action of allelic defense-related genes. For instance, expression of *WRKY45-1*, a susceptible allele of the rice transcription factor gene *WRKY45*, could generate a trans-acting TE-derived small interfering RNA, TE-siR815, to suppress the defense-related gene *siR815 Target 1* (*ST1*) by inducing RdDM and abolished the rice blight resistance mediated by *WRKY45-2*, a resistant allelic *WRKY45* (Table 1, Zhang et al., 2016). Similarly, DNA (de)methylation of two tandem miniature transposons (MITE1, MITE2) at the promoter of *PigmS*, a susceptible allele of the rice *Pigm* resistance genes, governs its transcription in a tissue-specific manner and thereby contributing to the balance of rice disease resistance and yield (Deng et al., 2017). These studies shed light on the important roles of DNA (de)methylation in the regulation of plant-pathogen interactions as well as its great potentials in crop disease resistance enhancement.

**Table 1:** Epigenetic processes and regulators involved in the crop-pathogen interactions.

**Epigenetic process category**	**Regulator gene name**	**Gene product family**	**Crop species**	**Involvement of gene product in crop- pathogen interactions and evidence**	**References**
DNA (de)methylation	*AeDRM2*	DNA methyltranferase functioning in *De novo* DNA methylation	*A. tauschii*	Knockdown of *AeDRM2* by BSMV-VIGS enhanced resistance of *A. tauschii* to the fungal pathogen *Bgt*.	Geng et al., 2019
	*OsDCL3*	Dicer-like endoribonuclease functioning in RdDM pathway	*O. sativa*	Infection of nematode *M. graminicola* was decreased in *Osdcl3a* and *Osdcl3b* mutants compared with the control.	Atighi et al., 2020
	*OsAGO4*	Argonaute protein functioning in RdDM pathway	*O. sativa*	Infection of *M. graminicola* was decreased in *Osago4a/b* mutant compared with the control.	Atighi et al., 2020
	*OsDRM2*	DNA methyltranferase functioning in *De novo* DNA methylation	*O. sativa*	Infection of *M. graminicola* was decreased in *Osdrm2* mutant compared with the control.	Atighi et al., 2020
	*OsDDM1*	A nucleosome-remodeling protein functioning in maintenance of DNA methylation	*O. sativa*	Infection of *M. graminicola* was decreased in *Osddm1* mutant compared with the control.	Atighi et al., 2020
	*OsTE-siR815*	Small interfering RNA functioning in RdDM pathway	*O. sativa*	OsTE-siR815 suppresses the defense-related gene siR815 Target 1 (ST1) by RdDM and abolished the rice blight resistance.	Zhang et al., 2016
Histone modifications	*TaGCN5*	Histone acetyltransferase	*T. aestivum*	TaGCN5 activates wheat cuticular wax biosynthesis required for triggering *Bgt* conidial germination.	Kong et al., 2020b
	*OsHDT701*	Histone deacetylase	*O. sativa*	OsHDT701 interacts with the rice RNase P subunit Rpp30 and negatively regulates rice defense responses to the fungal pathogen *M. oryzae* and bacterial pathogen Xoo by mediating histone deacetylation at PRR and defense genes.	Ding et al., 2012; Li et al., 2021
	*TaHDA6*	Histone deacetylase	*T. aestivum*	TaHDA6 functions in concert with WD40-repeat protein TaHOS15 and another HDAC TaHDT701 to suppress wheat defense responses to *Bgt* by reducing levels of histone acetylation at defense-related genes.	Liu et al., 2019; Zhi et al., 2020
	*TaSRT1*	Histone deacetylase	*T. aestivum*	Silencing *TaSRT1* by FoMV-VIGS significantly reduced CWMV infection in bead wheat.	Jin et al., 2020
	*OsJMJ704*	Histone demethylase	*O. sativa*	JMJ704 represses transcription of the rice defense negative regulator genes and positively regulates rice defense response against *Xoo* infection.	Hou et al., 2015
	*OsBRHIS1*	Monoubiquitinated histone binding protein	*O. sativa*	BRHIS1 represses the expression of some disease defense-related genes and rice blast resistance through interating with monoubiquitinated histone variants.	Li X. et al., 2015
Chromatin assembly and remodeling	*TaCHR729*	Chromatin remodeling factor	*T. aestivum*	TaCHR729 activates wheat cuticular wax biosynthesis required for triggering *Bgt* conidial germination.	Wang X. et al., 2019

## Histone Modifications And Their Multifaceted Functions In Crop Disease Resistance

As important epigenetic mechanisms, histone posttranslational modifications (PTMs) such as acetylation, methylation, and ubiquitylation usually occur at the histone N-terminal tails and get involved in the regulation of chromatin structures and functions (Ding and Wang, 2015; Ramirez-Prado et al., 2018a,b; Alonso et al., 2019; Wang C. et al., 2019). As a reversible process, histone acetylation is dynamically regulated by histone acetyltransferases (HATs) and histone deacetylases (HDACs) (Imhof and Wolffe, 1998). Generally, histone acetylation mediated by HATs could relax chromatin structure and facilitate gene transcription, whereas histone deacetylation mediated by HDACs contributes to gene repression (Song and Walley, 2016; Kong et al., 2020a). Exhaustive studies on Arabidopsis HATs (AtELP2 and AtELP3) and HDACs (AtHDA6, AtHDA19, AtSRT2, and AtHD2B) provide direct evidence for the involvement of histone (de)acetylation in the plant-pathogen interactions, which has been discussed in prior reviews (Kim et al., 2008; DeFraia et al., 2010; Defraia et al., 2013; Wang et al., 2010, 2015, 2017; Choi et al., 2012; Latrasse et al., 2017; Ramirez-Prado et al., 2018a,b). In addition, regulation of crop-pathogen interactions by histone (de)acetylation was supported by studies on the crop HATs and HDACs (Ding et al., 2012; Liu et al., 2019; Jin et al., 2020; Kong et al., 2020a; Zhi et al., 2020). For instance, wheat HAT complex TaGCN5-TaADA2 activates wheat wax biosynthesis by mediating histone acetylation at the promoters of biosynthesis-related genes, thereby providing wax signals for the conidial germination of fungal pathogen *Bgt* (Table 1, Kong et al., 2020b). Rice HDAC OsHDT701 interacts with the rice RNase P subunit Rpp30 and negatively regulates rice defense responses to the fungal pathogen *Magnaporthe oryzae* (*M. oryzae*) and bacterial pathogen Xoo by mediating histone deacetylation at PRR and defense genes (Table 1, Ding et al., 2012; Li et al., 2021). Similarly, wheat HDAC TaHDA6, the ortholog of Arabidopsis AtHDA6, could function in concert with WD40-repeat protein TaHOS15 and another HDAC TaHDT701 to suppress wheat defense responses to the fungal pathogen Bgt by reducing levels of histone acetylation at defense-related genes (Table 1, Liu et al., 2019; Zhi et al., 2020). In addition, a recent genome-wide identification and expression analysis of the *HDAC* gene family in bread wheat revealed that almost all *TaHDACs* were up-regulated by infection of the BSMV, Chinese wheat mosaic virus (CWMV), and wheat yellow mosaic virus (WYMV), suggesting their broad involvement in wheat defense response to viral infections (Jin et al., 2020). Significantly, silencing *TaSRT1*, a wheat HDAC gene, by Foxtail mosaic virus (FoMV)-based virus-induced gene silencing (VIGS) significantly reduced CWMV infection in bead wheat, indicating that *TaSRT1* contributes to the wheat susceptibility to CWMV infection (Table 1, Jin et al., 2020). In addition to these studies on crop HATs and HDACs, functional characterization of effectors secreted by crop pathogens also sheds light on the importance of histone (de)acetylation in the regulation of crop-pathogen interactions (Kong et al., 2017; Walley et al., 2018; Wang C. et al., 2019). For instance, PsAvh23, an effector secreted by the soybean oomycete pathogen *Phytophthora sojae*, could bind to the ADA2 subunit of the HAT complex SAGA and disrupt its assembly, and subsequently repress the activation of defense genes by disturbing the HAT complex-mediated H3K9 acetylation, thereby enhancing soybean susceptibility to the *P. sojae* infection (Kong et al., 2017). Similarly, effector molecule HC-toxin (HCT), an HDAC inhibitor produced by the fungal pathogen *Cochliobolus carbonum*, was found able to modulate plant HDACs to alter acetylation of plant histones and nonhistone proteins (Walley et al., 2018). It is well-known that plants employ methylation-mediated transcriptional gene silencing as an effective defense system against geminiviruses infection (Wang B. et al., 2018). Notably, V2 protein of Tomato yellow leaf curl virus (TYLCV) could interact with host histone deacetylase 6 (NbHDA6) and interfere with the recruitment of MET1 by HDA6, thereby suppressing methylation-mediated transcriptional gene silencing (Wang B. et al., 2018). In addition to regulating crop defense responses, histone (de)acetylation is essential to the regulation of pathogen growth and infection (Chen et al., 2018; He et al., 2018). For instance, MoSNT2 protein of *M. oryzae* could recruit the histone deacetylase complex to promote the histone H3 deacetylation at the promoter of autophagy genes *MoATG6, 15, 16*, and *22*, thereby regulating infection-associated autophagy (He et al., 2018). Another study revealed that wheat microbiome bacteria *Pseudomonas piscium* could secrete the compound phenazine-1-carboxamide to directly interfere with the activity of fungal histone acetyltransferase FgGcn5 and reduce the virulence of wheat pathogenic fungus *Fusarium graminearum* by altering histone acetylation (Chen et al., 2018). These studies revealed that histone (de)acetylation get involved in the regulation of many processes, from pathogen growth and infection to plant defense responses, in crop-pathogen interactions and provide new opportunity to control crop diseases.

Analogous to histone acetylation, histone methylation is a dynamic and reversible process co-regulated by histone methyltransferases and demethylases (Black et al., 2012). Unlike histone acetylation generally correlated with gene activation, histone methylation is associated with both gene activation and repression. For instance, H3K4 methylation and H3K36 methylation are important for active transcription, whereas H3K9 methylation and H3K27 methylation contribute to gene repression (Ding and Wang, 2015; Ramirez-Prado et al., 2018a,b; Wang C. et al., 2019). As extensively discussed in previous reviews, three Arabidopsis histone methyltransferases (AtATX1, AtSDG8, and AtSDG25) and two histone demethylase (AtJMJ27 and AtIBM1) have been reported to directly regulate plant-pathogen interactions (Alvarez-Venegas et al., 2007; Berr et al., 2010; Lee et al., 2016; Dutta et al., 2017; Chan and Zimmerli, 2019; Zhang et al., 2020). In addition, increasing evidence supported that histone (de)methylation plays key role in regulating crop-pathogen interactions (Hou et al., 2015; Meller et al., 2018). For instance, spraying potato leaves with β-aminobutyric acid (BABA), a non-protein amino acid effective in improving plant disease resistance, could increase deposition of histone marks H3K4me2 and H3K27me3 on *NPR1* and *SNI1*, regulatory genes in systemic acquired resistance (SAR), thereby reprogramming responsiveness of the defense-related genes (*PR1* and *PR2*) and contributing to the potato intergenerational resistance to *P. infestans* (Meller et al., 2018). Jumonji C (JmjC) domain-containing proteins generally function as histone lysine demethylases. Genome-wide identification and expression analysis of the rice *JmjC* gene showed that expressions of 15 *JmjC*s were induced by infection of bacterial blight pathogen *Xoo*. Further studies revealed that JMJ704 represses transcription of the rice defense negative regulator genes such as *NRR, Os-11N3*, and *OsWRKY62* by reducing H3K4me2/3 at promoters of these genes, thereby potentiating rice defense response against *Xoo* infection (Table 1, Hou et al., 2015).

Although less explored compared with histone acetylation and methylation, histone mono-ubiquitination primarily occurs on histone H2A and H2B, and plays an important role in transcriptional regulation. In Arabidopsis, histone H2B mono-ubiquitination (H2Bub1) is mediated by the ubiquitin ligases HUB1 and HUB2 (Dhawan et al., 2009; Hu et al., 2014; Zou et al., 2014; Zhao et al., 2020). Functional characterization of HUB1 and HUB2 revealed that H2Bub1 contributes to the Arabidopsis resistance against bacterial pathogen *Pst* DC3000 and fungal pathogen *Verticillium dahliae* (*Vd*) by potentiating transcription of resistance genes (*SNC1* and *RPP4*) and modulating the dynamics of cortical microtubules (MTs) (Hu et al., 2014; Zou et al., 2014). Interestingly, HUB1 was revealed to interact with MED21, a subunit of the Arabidopsis Mediator, and positively regulates defense against necrotrophic fungal pathogens *Botrytis cinerea* and *Alternaria brassicicola* in Arabidopsis (Dhawan et al., 2009). In addition, a recent study demonstrated that HUB-mediated H2Bub1 positively regulates the expression of the NADPH oxidase RbohD, a critical defense modulator, by enhancing the H3K4me3 enrichment, indicating the complex interplays among histone mono-ubiquitination, methylation, and mediator complex in the regulation of plant defense responses (Zhao et al., 2020). In addition to HUBs, binding proteins of monoubiquitinated histone are also demonstrated to be involved in the regulation of plant defense response (Li X. et al., 2015). For instance, the rice SWI/SNF2 ATPase BRHIS1-containing complex was found to repress the expression of some disease defense-related genes (*OsPBZc* and *OsSIRK1*), as well as rice blast resistance, through specific interaction with monoubiquitinated histone variants H2B.7 and H2A.Xa/H2A.Xb/H2A.3, in the absence of pathogen infection (Table 1, Li X. et al., 2015). Characterizing more HUBs and monoubiquitinated histone binding proteins in crops will improve our understanding of the multiple roles of histone mono-ubiquitination in regulating crop-pathogen interactions in future research.

## The Regulatory Roles of Chromatin Assembly and Remodeling in Plant Disease Resistance

Chromatin structure is essential to the organization of plant genomes and is dynamically controlled by histones and a wide range of modulators. In the regulation of gene transcription, chromatin structure governs the DNA accessibility to transcription machinery and plays a vital role in transcriptional regulation (Li et al., 2007). Increasing evidence revealed that chromatin structure gets involved in the regulation of plant defense responses (Walley et al., 2008; Johnson et al., 2015; Mozgová et al., 2015; Muñoz-Viana et al., 2017; Wang C. et al., 2019; Yang et al., 2019). For instance, knockdown of histone H2B in *Nicotiana benthamiana* using Tobacco rattle virus (TRV)-based virus-induced gene silencing (VIGS) resulted in the up-regulation of SA biosynthesis/signaling-related genes such as *EDS1, ICS1*, and *NPR1*, leading to the increased endogenous SA accumulation and enhanced resistance against potato virus X (PVX) infection (Yang et al., 2019). CHROMATIN ASSEMBLY FACTOR 1 (CAF-1) is an evolutionarily conserved histone chaperone essential for post-replicative *de novo* assembly of histones into nucleosomes (Mozgová et al., 2015; Muñoz-Viana et al., 2017). In Arabidopsis, the absence of CAF-1 resulted in the reduced nucleosome occupancy and high H3K4me3 at defense response genes *PR1, PR5, WRKY6*, and *WRKY53*, leading to spurious activation of SA-dependent defense response in plants grown under standard non-sterile growth conditions, further confirming the repressive role of chromatin assembly in the transcription of defense-related genes (Mozgová et al., 2015; Muñoz-Viana et al., 2017).

In addition to histone H2B and histone chaperon CAF-1, chromatin remodeling factors that could use the energy from ATP hydrolysis to disrupt the DNA-histone association also get involved in the regulation of plant defense responses (Walley et al., 2008; Johnson et al., 2015; Berriri et al., 2016; Wang C. et al., 2019; Huang et al., 2021). For instance, SWI/SNF chromatin remodeling protein SYD (SPLAYED) was revealed to negatively regulate the transcription of Arabidopsis R gene *SNC1* and suppress the *SNC1*-mediated resistance to the bacterial pathogen *Pseudomonas syringae* pv. *maculicola* ES4326 (Walley et al., 2008; Johnson et al., 2015). SWP73A, an Arabidopsis ortholog of the mammalian SWI/SNF chromatin remodeling protein BAF60, was found to directly bind to the promoters of resistance genes *RPS2* and *RPS4* and suppress their expressions (Huang et al., 2021). In addition, SWP73A could suppress the transcription of RNA splicing regulator gene *CDC5* and affect the alternative splicing of *RPS2* and *RPS4*, thereby suppressing Arabidopsis defense responses (Huang et al., 2021). Interestingly, although SWR1 chromatin-remodeling complex (SWR1c) catalyzes the replacement of histone H2A by the histone variant H2A.Z in nucleosomes in eukaryotic gene regulation, SWR1c subunits and H2A.Z were revealed to have non-overlapping functions in plant immunity and gene regulation (Berriri et al., 2016). At the same time, the regulation of crop disease resistance by chromatin remodeling proteins was supported by emerging evidence (Wang X. et al., 2019). For instance, the wheat CHD3-type chromatin remodeling protein TaCHR729 was reported to bind to the promoter regions of wheat wax biosynthesis genes *3-KETOACYL-CoA SYNTHASE* (*TaKCS6*), and positively regulate the *TaKCS6* transcription by enhancing the permissive epigenetic mark H3K4me3 at the promoter region of *TaKCS6*. Silencing of *TaCHR729* by BSMV-VIGS could down-regulate the wheat wax biosynthesis and reduce conidial germination of *Bgt*, suggesting that the chromatin remodeling factors TaCHR729 contributes to the wheat-powdery mildew interaction through epigenetic activation of wheat cuticular wax biosynthesis (Table 1, Wang X. et al., 2019).

## Involvement Of Epigenetic Memory In Defense Priming

In crop protection, treatment of crop plants with plant pathogens or priming agents such as salicylic acid (SA), acibenzolar S-methyl (BTH), β-aminobutyric acid (BABA), methyl-jasmonic acid (MeJA), pipecolic acid (Pip), and azelaic acid (AzA) could lead to a primed state of defense responses, as well as systemic acquired resistance (SAR), to future pathogen challenges (Reimer-Michalski and Conrath, 2016; Mauch-Mani et al., 2017). These defense priming and SAR could be sustained over generations (Jaskiewicz et al., 2011; Luna et al., 2012; Slaughter et al., 2012; Iwasaki and Paszkowski, 2014). For instance, treatment of common bean (*Phaseolus vulgaris* L.) with priming agent BABA could enhance plant disease resistance against the bacterial pathogen *Pseudomonas syringae* pv. Phaseolicola (Ramírez-Carrasco et al., 2017). Notably, expression of the defense-related *PvPR1* gene (*Phaseolus vulgaris PR1*, the common bean ortholog of Arabidopsis *PR1-1*) exhibited a priming response against pathogen infections for at least two generations (Ramírez-Carrasco et al., 2017). In addition, epigenetic profiling revealed that this defense priming is generally associated with DNA methylation and histone modifications (Lämke and Bäurle, 2017; He and Li, 2018). For instance, treatment of Arabidopsis leaves with SA synthetic analog BTH or bacterial pathogen *Pseudomonas syringae* pv. Maculicola could induce enhancement of permissive epigenetic mark H3K4me2/3 on defense gene promoters, which is supposed to prepare chromatins for rapid activation of defense genes upon pathogen re-infections (Slaughter et al., 2012). Similarly, defense priming induced by MeJA in rice could enhance the levels of H3K4me3 and H3K9ac at the promoters of defense-related gene *OsBBPI*, and affect the genome-wide DNA methylation (5 mC) levels, leading to a chromatin-based memory of wounding stress (Laura et al., 2018).

Increasing evidence revealed that DNA methylation and histone modifications get involved in transgenerational defense priming (Slaughter et al., 2012; He and Li, 2018; Stassen et al., 2018; Sharrock and Sun, 2020). For instance, SAR induced by bacterial pathogen *Pst*DC3000 could be inherited to generate progeny (P1) exhibiting potentiated expression of SA-inducible defense-related genes such as *PR1, WRKY6*, and *WRKY53*, as well as enhanced resistance against hemibiotrophic bacterial pathogen *Pst*DC3000 and biotrophic oomycete pathogen *Hpa* (Luna et al., 2012; Slaughter et al., 2012). At the same time, these P1 progeny exhibited reduced expression of JA-inducible defense-related genes such as *PDF1.2* and *VSP2*, as well as decreased resistance against necrotrophic fungal pathogen *Alternaria brassicicola* (Luna et al., 2012). Further studies revealed that this difference in the expression of SA- and JA-inducible defense-related genes in P1 progeny was not caused by hormone level changes but associated with shifts in histone modifications H3K27me3 and H3K9ac at the promoters of these defense-related genes (Luna et al., 2012). In addition, priming with bacterial pathogen *Pst*DC3000 resulted in DNA hypomethylation in Arabidopsis, and DNA hypomethylation mutant *drm1drm2cmt3* could mimic this transgenerational SAR phenotype, further confirming that this transgenerational defense priming in the systemic plant immune response is governed by DNA methylation and histone modifications (Luna et al., 2012).

## Application Of Epigenetic Variations In Crop Disease Resistance Improvement

Intensive monoculture of domesticated crops on fertilized land increases the prevalence and incidence of plant diseases, which became severe under more frequent extreme weathers resulting from climate changes such as drought and heat weaves (Bruce, 2012; Cohen and Leach, 2020; Zytynska et al., 2020). Even with crop-protection strategies such as conventional chemical control and eco-friendly biological control, plant diseases account for approximately 20% global yield loss in important crops (Nicaise, 2017; Oliva and Quibod, 2017; Silva et al., 2018). Due to the rapid evolution of plant pathogens, classical breeding approaches for crop disease resistance improvement, which mainly rely on the exploitation of resistance (R) genes, have become less effective (Rodriguez-Moreno et al., 2017). In addition, intensive artificial selection in the conventional breeding practices has eroded the genetic diversity of R genes (Rodriguez-Moreno et al., 2017). Cross-breeding crop varieties with their wild relatives and developing genetically modified organisms (GMOs) represent new promising approaches in crop resistance breeding (Bruce, 2012). As an alternative direction, epigenetic processes such as DNA methylation, histone modifications, chromatin assembly and remodeling could respond to pathogen infections and broaden phenotypic diversity suitable for crop resistance improvement. Therefore, epi-breeding referring to the genetic breeding for epigenetic changes provides new avenues for crop resistance improvement (Gallusci et al., 2017; Springer and Schmitz, 2017; Latutrie et al., 2019; Tirnaz and Batley, 2019; Varotto et al., 2020).

Both natural epigenetic diversity and artificially induced epigenetic variations could influence plant disease resistance and have great potentials in epi-breeding for crop resistance improvement. For instance, inheritable natural epialleles associated with plant development and stress adaptation have been characterized in some model and crop plants such as Arabidopsis, rice, oilseed rape, apple, tomato, and melon (Cubas et al., 1999; Manning et al., 2006; Martin et al., 2009; Long et al., 2011; Telias et al., 2011; Chen and Zhou, 2013; Liu et al., 2015; Latutrie et al., 2019). As discussed in the DNA (de)methylation section in detail, DNA hypomethylation was usually induced by biotic and abiotic stresses, suggesting that these stress conditions could be employed to induce disease-resistance-related epigenetic variations in crop plants. Furthermore, epigenetic variants could be experimentally obtained by chemicals treatments, mutations in epigenetic machinery, induced gene-specific DNA methylation, and epigenome editing (Gallusci et al., 2017; Springer and Schmitz, 2017; Latutrie et al., 2019; Tirnaz and Batley, 2019; Varotto et al., 2020). For instance, 5-azacytidine and zebularine, two non-methylable cytosine analogs, are widely used as inhibitor of DNA methyltransferases to chemically induce DNA demethylation (Baubec et al., 2009; Griffin et al., 2016). Although epigenetic changes induced by these stress conditions and chemical treatments are transient, alteration of epigenetic regulation could result in the mobilization of TE and formation of heritable epialleles, which could be employed for breeding purposes (Mirouze and Paszkowski, 2011). Through mutagenizing DNA (de)methylation machinery and crossing genetically identical plants with different DNA methylation levels, epigenetic recombinant inbred line (epiRIL) populations with similar genomic backgrounds but different DNA methylation levels at specific chromatin regions were successfully created in Arabidopsis, and these epiRIL populations displayed discernible phenotypic variations such as altered disease resistance (Zhang et al., 2018). However, creating epiRILs in crop plants such as bread wheat and maize has proved more challenging due to lack of DNA (de)methylation mutants, low genome stability, as well as low rates of transformation, regeneration and mutagenesis in crop plants (Kapazoglou et al., 2018).

Induced gene-specific DNA methylation and epigenome editing represent new promising approaches to generate epiallelic variations in model and crop plants. Indeed, 24-nt short-interfering RNAs (siRNAs) generated by double-stranded RNAs (dsRNAs) could direct DNA methylation and induce transgenerationally heritable silencing of plant endogenous genes (Kasai and Kanazawa, 2013; Dalakouras and Papadopoulou, 2020). Notably, exogenous application of dsRNA designed to target gene promoter regions resulted into promoter RdDM, which could circumvent crop breeding obstacles such as low transformation and regeneration rate, thereby representing a novel methodology for crop epigenome modification (Dalakouras and Papadopoulou, 2020; Dalakouras and Ganopoulos, 2021). In mammalian cells, epigenome editings have been established through fusing an inactive variant of Cas9 (CRISPR associated protein 9), the most widely used sequence-specific nucleases (SSNs) in the CRISPR system (clustered regularly interspaced short palindromic repeat), to histone modifying enzymes such as histone demethylase LSD1, DNA methyltransferase DNMT3A and the catalytic core of the human histone acetyltransferase p300 (Hilton et al., 2015; Kearns et al., 2015; Kungulovski and Jeltsch, 2016; Vojta et al., 2016; Liu and Moschou, 2018). Similarly, Johnson et al. selectively targeted regions of Arabidopsis genomes for DNA methylation through fusing a zinc finger (ZF) peptide with the RdDM component SUVH9, which paves the path to the locus-specific epigenome editing in crop plants (Kolb et al., 2005; Johnson et al., 2014). However, to achieve a similar CRISPR or ZF-based epigenome editing in crop plants, it necessitates either stable transformation of crop plants with corresponding CRISPR or ZF elements or transfection of crop protoplasts with preassembled CRISPR or ZF complex, which is obstructed by low rates of transformation and regeneration in some crop plants such as bread wheat (Woo et al., 2015). In addition, placing crop products improved by epigenome modification techniques on the market also requires clarification and update on the current regulatory framework applied for GMOs and GEENs (genome editing using engineered endonuclease) in some countries (Metje-Sprink et al., 2019).

As important questions in epi-breeding, transmission features of epigenetic variations such as stability and inheritability should be discussed. It was widely demonstrated that differentially methylated regions (DMRs) in epialleles are transmitted in a Mendelian manner during meiosis, indicating that DNA methylation could function as a stable and heritable epigenetic mark over generations (Schmitz et al., 2013; Li Q. et al., 2015). As demonstrated by elegant studies in plant vernalization, histone modifications could be stably transmitted through mitosis (Baulcombe and Dean, 2014). For instance, enrichment of repressive epigenetic mark H3K27me3 at the *FLC* locus, as well as the *FLC* silencing, induced upon cold exposure could be stably maintained during the rest life cycle of Arabidopsis (Coustham et al., 2012). Although the involvement of histone modifications in transgenerational stress memory has been supported by a few studies, the transgenerational inheritance of histone modifications is rather limited. For instance, vernalization involving H3K27 methylation is reset in each generation (Baulcombe and Dean, 2014). Therefore, histone modifications likely could not be stably transmitted during sexual reproduction.

Epi-breeding strategies for crop resistance improvement are determined by both transmission features of epigenetic variations and propagation types of crop plants (Gallusci et al., 2017; Latutrie et al., 2019). As epigenetic marks stably transmitted across plant generations, DNA methylation could be used in breeding crop plants propagated vegetatively or by seed. In contrast, histone modifications could not be transgenerationally inherited and are only suitable for breeding clonally propagated crops. As an efficient large-scale reproduction strategy, clonal propagation has an advantage in fixing beneficial epigenetic traits and is applicable for over 60% of crops, including potatoes, yams, taros, sorghums, and cassavas (Meyer et al., 2012; Latutrie et al., 2019). In addition, molecular epigenomic markers such as differentially methylated regions (DMRs) could be used for the early characterization of epigenetic states in meristem/seed screening and contributes to the marker-assisted selection in crop epi-breeding for disease resistance improvement (Gallusci et al., 2017; Latutrie et al., 2019).

Epigenetic modeling could predict the effect of epigenetic variations on plant phenotype and fill the gap between epigenetic variations and epi-breeding for disease resistance improvement (Gallusci et al., 2017). As discussed in prior reviews, a statistical model could be used to explore the linkage between DNA methylomes and transcriptomes without any knowledge on underlying mechanisms of biological processes, whereas a more advanced process-based model was developed to predict the effects of epigenetic variations on gene expression, as well as plant phenotypes, through employing equations defining the essence of well-studied biological processes (Richards et al., 2012; Buck-Sorlin, 2013; Gallusci et al., 2017). For instance, statistical models were employed to identify novel relationships between DNA methylation and gene expression in *Mimulus guttatus* and link DNA methylation information with plant height variances in *A. thaliana* (Colicchio et al., 2015; Hu et al., 2015). Similarly, process-based models were developed to link histone modifications, gene expression, and plant phenotypes such as vernalization and lycopene metabolism in Arabidopsis and tomato (Angel et al., 2011; Gallusci et al., 2017). Therefore, epigenetic modeling, especially the process-based model, could be used to investigate the effect of specific epigenetic variations on plant disease resistance and guide the decision to induce or suppress these epigenetic variations for resistance improvement in crop epi-breeding.

## Concluding Remarks And Perspectives

In this review, we highlighted the importance of epigenetic processes in the regulation of crop disease resistance, and discussed the potentials, challenges, and strategies of exploiting epigenetic variation for crop disease resistance improvement. Epigenetic processes such as DNA methylation, histone post-translational modifications, chromatin assembly and remodeling are highly interconnected and orchestrate plant transcriptional reprogramming in biotic and abiotic stress responses. In addition to modulating defense-related transcription essential for generational and transgenerational defense priming, epigenetic variations respond to pathogen infections and could be harnessed by pathogenic effectors, thereby increasing plant phenotypic plasticity and securing crop production under pathogen challenges.

**Figure 1:**
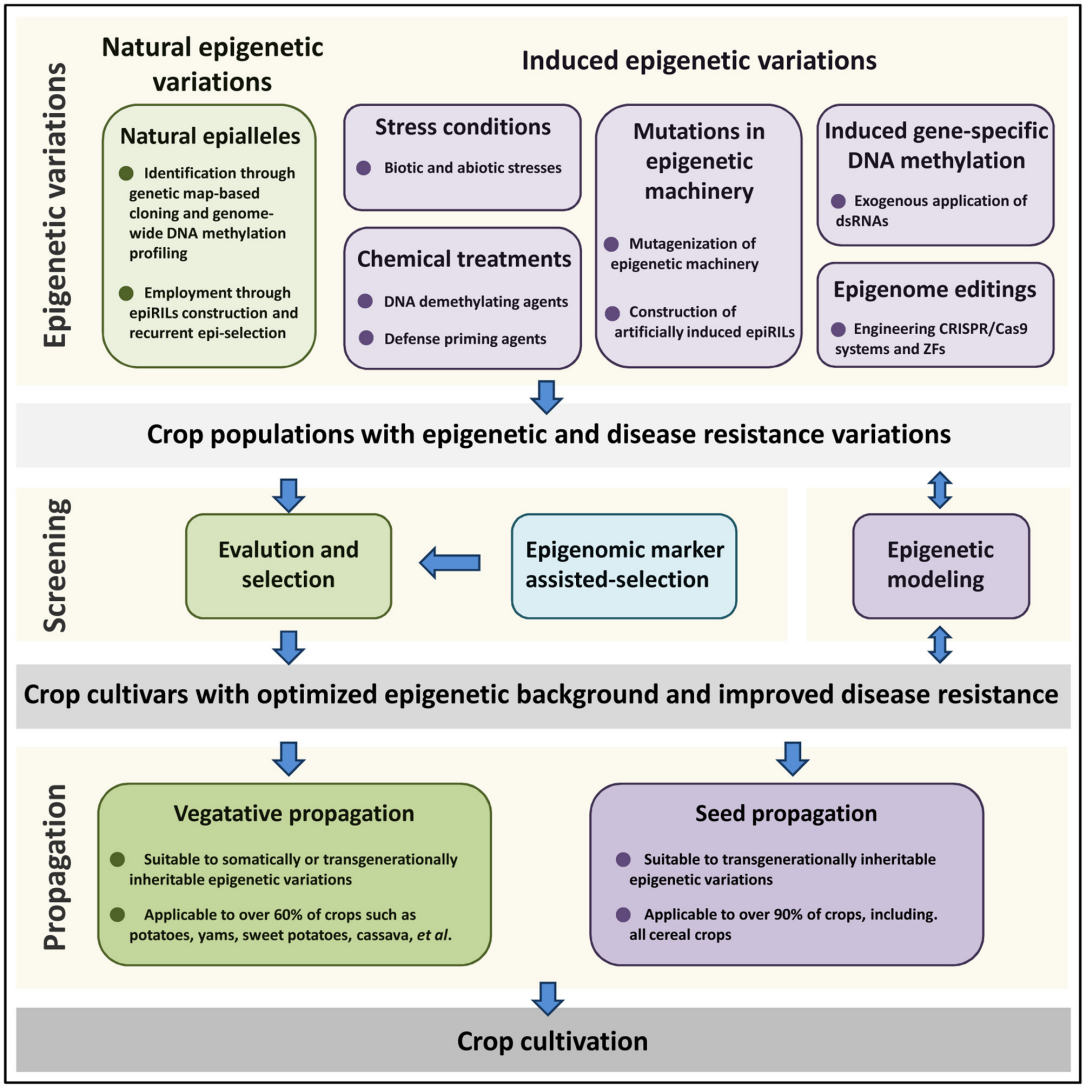
Epi-breeding design for crop disease resistance improvement. Epigenetic variations are either derived from natural populations, or induced by stresses, chemical treatments, mutations in epigenetic machinery, induced gene-specific DNA methylation, and epigenome editings. After epigenetic variations have been generated, crop variants with improved disease resistance are chosen and propagated. DNA methylation marks can be transgenerationally inherited and suitable for epi-breeding in all kinds of crops. However, histone post-translational modifications are only relevant to epi-breeding in clonally propagated crops. Epigenomic variations such as DMRs related to disease resistance traits could be employed as molecular epigenomic markers to assist the evaluation and selection process. In addition, epigenetic modeling could be used to predict the effect of epigenetic variations on crop disease resistance and provide instruction for crop epi-breeding design.

As shown in Figure 1, both natural epigenetic diversity and epigenetic variations artificially induced by stress conditions, chemical treatments, mutations in epigenetic machinery, induced gene-specific DNA methylation and epigenome editing, could contribute to crop epi-breeding for disease resistance improvement. DNA methylation marks can be transgenerationally inherited and suitable for epi-breeding in all crops. However, information about histone post-translational modifications is likely to be erased during meiosis and only relevant to epi-breeding in clonally propagated crops. Since over 50% of seed-propagated crops can be clonally propagated, clonal propagation strategies such as root cutting, grafting, air layering, and tissue culture might provide a great opportunity for the development of crop epi-breeding for disease resistance improvement. Epigenomic variations such as DMRs related to disease resistance traits could be employed as molecular epigenomic markers to assist the evaluation and selection processes. In addition, epigenetic modeling could be used to predict the effect of epigenetic variations on crop disease resistance and provide instruction for epigenetic engineering in crop resistance breeding. With the development of epigenetic methodology and theory on crop-pathogen interactions, exploiting epigenetic variations would provide new avenues for crop disease resistance improvement in the future.

## Author Contributions

PZ and CC wrote the manuscript. Both authors contributed to the article and approved the submitted version.

## Conflict of Interest

The authors declare that the research was conducted in the absence of any commercial or financial relationships that could be construed as a potential conflict of interest.
